# Advanced membrane‐based electrode engineering toward efficient and durable water electrolysis and cost‐effective seawater electrolysis in membrane electrolyzers

**DOI:** 10.1002/EXP.20220112

**Published:** 2023-10-20

**Authors:** Jiayi Tang, Chao Su, Zongping Shao

**Affiliations:** ^1^ WA School of Mines: Minerals, Energy and Chemical Engineering (WASM‐MECE) Curtin University Perth Western Australia Australia; ^2^ School of Energy and Power Jiangsu University of Science and Technology Zhenjiang China

**Keywords:** anion exchange membrane water electrolyzers, direct seawater electrolysis, electrode engineering, membrane electrode assembly, proton exchange membrane water electrolyzers

## Abstract

Researchers have been seeking for the most technically‐economical water electrolysis technology for entering the next‐stage of industrial amplification for large‐scale green hydrogen production. Various membrane‐based electrolyzers have been developed to improve electric‐efficiency, reduce the use of precious metals, enhance stability, and possibly realize direct seawater electrolysis. While electrode engineering is the key to approaching these goals by bridging the gap between catalysts design and electrolyzers development, nevertheless, as an emerging field, has not yet been systematically analyzed. Herein, this review is organized to comprehensively discuss the recent progresses of electrode engineering that have been made toward advanced membrane‐based electrolyzers. For the commercialized or near‐commercialized membrane electrolyzer technologies, the electrode material design principles are interpreted and the interface engineering that have been put forward to improve catalytic sites utilization and reduce precious metal loading is summarized. Given the pressing issues of electrolyzer cost reduction and efficiency improvement, the electrode structure engineering toward applying precious metal free electrocatalysts is highlighted and sufficient accessible sites within the thick catalyst layers with rational electrode architectures and effective ions/mass transport interfaces are enabled. In addition, this review also discusses the innovative ways as proposed to break the barriers of current membrane electrolyzers, including the adjustments of electrode reaction environment, and the feasible cell‐voltage‐breakdown strategies for durable direct seawater electrolysis. Hopefully, this review may provide insightful information of membrane‐based electrode engineering and inspire the future development of advanced membrane electrolyzer technologies for cost‐effective green hydrogen production.

## Graphical abstract

This review summarizes the cutting‐edge electrode engineering strategies, including electrode structure engineering, interface engineering, catalyst utilization improvement, electrode reaction environment regulation, cell‐voltage‐breakdown strategy, and so on toward the most promising commercialized, near‐commercialized, and advanced membrane water electrolyzer technologies, aiming to achieve efficient, durable, and economical green hydrogen production through water electrolysis.

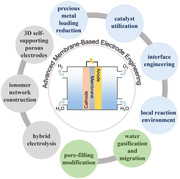

## 1 INTRODUCTION

Electrochemical water splitting for green hydrogen production is entering a new stage of industrialization, thus reducing components and operation costs, improving efficiency, and enhancing stability are undoubtedly the most critical three issues to push forward the large‐scale manufacture and extensive use of clean hydrogen energy.^[^ ^1^, ^2^ ^]^ Compared to traditional alkaline electrolysis cells for hydrogen production at a relatively low electric efficiency of 50%‒60% with low gas product purity, polymer electrolyte membrane‐based electrolyzers, including proton exchange membrane water electrolyzer (PEMWE) and anion exchange membrane water electrolyzer (AEMWE) have gained noticeable popularity.^[^ ^3^, ^4^ ^]^ Owing to their unique membrane‐based zero‐gap configurations, a more acceptable electric efficiency of 70%‒80% has been achieved with high‐purity products. The overall water electrolysis efficiency in polymer electrolyte membrane electrolyzers is largely restricted by the anodic oxygen evolution reaction (OER), due to the apparently higher anodic overpotential compared with that required for the cathodic hydrogen evolution reaction (HER).^[^ ^5^ ^]^ Although a burgeoning bipolar membrane is proposed with the implementation of a bipolar interface to create more favorable acidic HER and alkaline OER reaction environments, but this area is still in a nascent state with more research focus on membrane science,^[^ ^6^, ^7^, ^8^ ^]^ and the cell voltage breakdown strategies with bipolar membrane have not been established yet.^[^ ^9^ ^]^ Therefore, researchers are keen on exploring advanced membrane‐based water electrolyzer technologies. From one perspective, the cost reduction and efficiency improvement are expected to be achieved by addressing the existing issues with PEMWE and AEMWE. From another perspective, more universal and industrially‐applicable electrolyzer technologies are under investigation to make breakthroughs in pure water electrolysis, and likely achieve direct seawater electrolysis.

Membrane electrode assembly (MEA) is the core of membrane water electrolyzers.^[^ ^10^, ^11^ ^]^ Although for the electrocatalyst applied in MEA, its intrinsic activity and stability in principle determine the reaction performance on the electrode, however, capability of the electrocatalyst is commonly not fully expressed on electrodes, especially for device‐level amplified electrodes targeting practical applications. Therefore, electrode engineering, which includes structure engineering and interface engineering, is by no means a trivial issue for further exploring the practical application of the electrocatalysts. Concretely, the electrode structure control, generally involving the catalyst layer thickness, porous architecture, and the distribution of catalytic sites, plays a critical role in enabling sufficient accessible active sites. While the interface engineering regulation, involving the electronic and ionic conduction during electrode reaction, mainly affects the effective utilization of catalytic sites.

For aforementioned membrane‐based electrolyzers, electrode engineering also involves the adjustment of reaction environment,^[^ ^12^ ^]^ the voltage breakdown strategy,^[^ ^13^, ^14^ ^]^ and the combination of advanced membrane technology to achieve highly‐efficient, durable, scalable, and possible cost‐effective water electrolysis with impure water.^[^ ^15^ ^]^ Therefore, electrode engineering in recent years has gained a noticeable research popularity and been considered to demonstrate a promising lead to rectify the limitations for green hydrogen production through water electrolysis.

**FIGURE 1:**
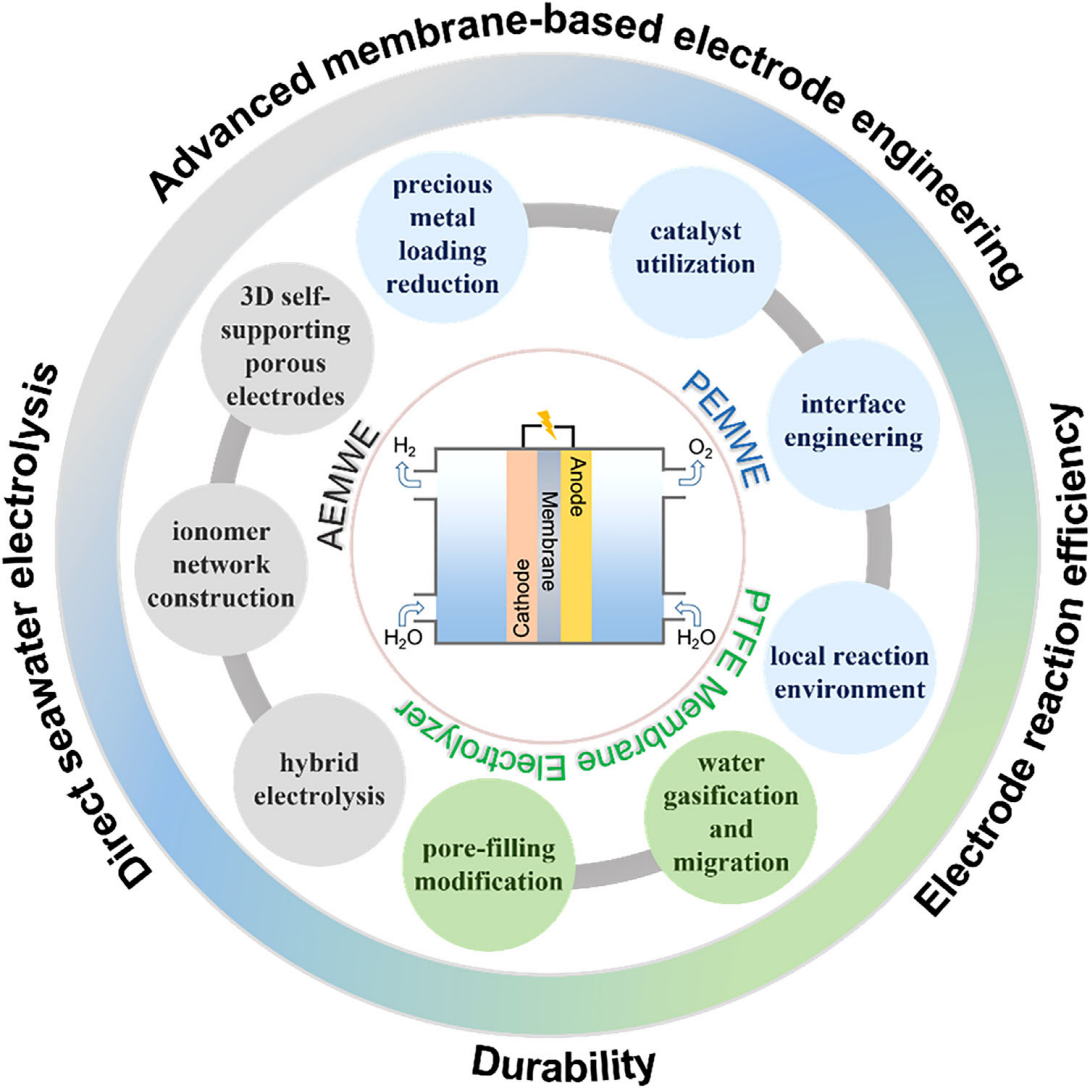
A schematic overview of the advanced membrane‐based electrode engineering toward efficient and durable water electrolysis, as well as direct seawater electrolysis.

Herein, we present an overview of cutting‐edge electrode engineering toward membrane‐based electrolyzer technologies for water electrolysis. Different from the previous reviews, either focused on summarizing the development of anodic OER and cathodic HER catalysts involving acidic and alkaline operating conditions,^[^ ^16^, ^17^, ^18^, ^19^ ^]^ or dedicated to review the status and perspectives of various membrane‐based electrolyzer technologies,^[^ ^20^, ^21^ ^]^ this review provides insightful information on applying the available catalysts in MEAs to achieve highly efficient, durable, and energy‐saving water electrolysis. As illustrated in Figure 1, recent advances achieved with commercially promising polymer electrolyte membrane electrolyzers, such as PEMWEs and AEMWEs, as well as other cutting‐edge membrane electrolyzers as innovative next‐generation technology are comprehensively covered in this review. For the commercialized PEMWE, we summarize the recent progress in reducing the precious metal loading, improving catalytic site utilization, optimizing the electrode interfaces, and adjusting the reaction environment, given the pressing issues of cost reduction and efficiency improvement. For the AEMWE under R&D state, we highlight the electrode engineering toward using precious metal free electrocatalysts to build rational electrode architectures and effective reaction interfaces. Besides, a feasible cell‐voltage‐breakdown strategy in an alkaline environment, such as hybrid electrolysis is discussed to push forward the commercialization of AEMWE. In addition, this review also discussed the innovative ways as proposed to break the barriers of current electrolyzers, and realize direct seawater electrolysis, for example, by applying a more durable polytetrafluoroethylene (PTFE) membrane and the possible modification of the PTFE membrane for water electrolysis. Hopefully, this review may bridge the gap between catalyst/electrode development and the industrialization of hydrogen production through water electrolysis.

## 2 ELECTRODE ENGINEERING TOWARD PEMWES

PEMWE, since its first inception in the 1960s by General Electric, has been developed into the second commercialized water electrolysis technology toward large‐scale hydrogen production besides the alkaline electrolysis cell.^[^ ^22^ ^]^ Extensive research interests have been paid to the PEM electrolyzer/stack assembly,^[^ ^23^ ^]^ mass transport optimization,^[^ ^24^ ^]^ system and auxiliary system design for promoting large‐scale manufacture,^[^ ^25^, ^26^ ^]^ while electrode engineering involving multi‐scale factors is still the key to cost‐effectiveness and energy efficiency. For the fabrication of electrodes in PEMWEs, the catalyst‐coated‐membrane (CCM) technique has been commonly used and is considered the mainstream way for electrode mass production. Several advantages of the CCM technique have made this process more popular than other electrode fabrication techniques, such as the catalyst‐coated‐substrate (CCS) method,^[^ ^27^ ^]^ and more suitable for the PEMWE operating conditions. Generally, the CCM approach enables close contact between the catalyst layer and the membrane, and this is critical to reduce the inner ohmic resistance of the electrolyzer configuration and improve the water electrolysis efficiency.^[^ ^28^ ^]^ Advanced bottom‐up strategy was also proposed to enable even uniform distribution of ionomer for forming more efficient catalyst‐ionomer interface.^[^ ^29^ ^]^ Another merit of the CCM approach lies in its feasibility to make ultra‐thin catalyst layers, which is favorable to reduce the catalyst loading.^[^ ^30^, ^31^, ^32^ ^]^ However, since every coin has two sides, the closely membrane‐attached catalyst layer could suffer from cracking, delamination, or debonding due to the thermal and mechanical strain of the membrane during electrode reactions,^[^ ^33^, ^34^ ^]^ while the membrane is also likely to be poisoned by the metal ions dissolved in the catalysts.^[^ ^35^, ^36^ ^]^ For the OER electrode in PEMWEs accompanied by water erosion during the operation, this drawback could be one of the most important failure mechanisms of the electrode. In this chapter, we are going to summarize the recent progress on electrode engineering for PEMWEs, aiming to reduce the precious metal loading, and improve the catalytic site utilization, enhance electrode structural and functional stability, as well as electrode durability in impure water (such as seawater).

### 2.1 Toward reducing precious metal loading: PEMWE applicable catalyst design

At present, iridium (Ir)‐ and ruthenium (Ru)‐based oxide materials, such as IrO_2_, Ir*_x_* Ru_1−_ *_x_* O_2_, and Ir black, are still considered the mainstream catalysts for the acidic OER in commercial PEMWEs due to their superior activity and desirable stability.^[^ ^37^, ^38^, ^39^ ^]^ Ir is one of the scarcest metals on earth, with a low abundance in the Earth's crust of about 0.001 parts per million, which makes it hard to reduce the capital cost of large‐scale PEMWE, with IrO_2_ as the only viable commercial catalyst at present. Besides, a high catalyst loading of the OER electrode is generally required to achieve satisfactory electrolysis efficiency due to the sluggish kinetics of the acidic OER. While for the HER electrode, although Pt/C is used as the most‐effective catalyst, an ultra‐low Pt loading of less than 0.05 mg cm^‒2^ is achievable due to the fast HER kinetics.^[^ ^40^ ^]^ Although the development of non‐precious metal‐based electrocatalysts for the acidic OER is an attractive research area at present, with some innovatively designed materials come to the fore,^[^ ^41^, ^42^ ^]^ for example, CoMn‐based spinel oxide,^[^ ^43^ ^]^ and MnSb‐based rutile system.^[^ ^44^ ^]^ However, the practical application of these materials still requires prudent two‐pronged theoretical and experimental verification under PEMWE operating conditions due to the long‐standing elusiveness of their intrinsic electronic conductivity and actual stability.

**FIGURE 2:**
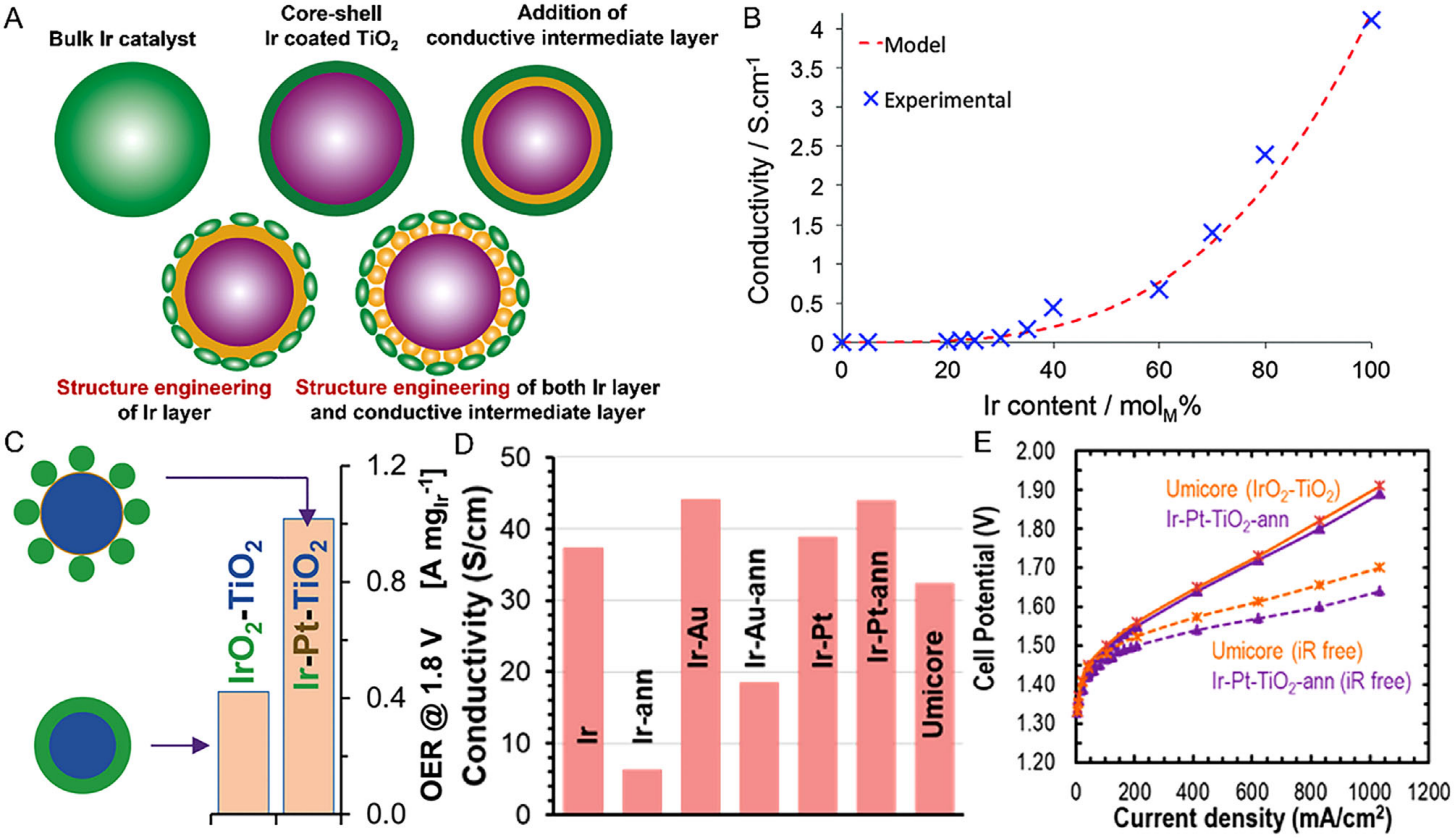
Strategies toward reducing the precious metal (Ir or Ru) loading. (A) Schematic illustration of bulk Ir catalyst and architecture engineering of supported Ir catalysts.^[^ ^47^ ^]^ (B) Experimental and modeled electrical conductivity trends of IrO_2_ deposited TiO_2_ nanoparticle versus Ir content.^[^ ^56^ ^]^ (C) Comparison of the OER mass activity of two TiO_2_ supported Ir catalysts. (D) Comparison of the electronic conductivities of various TiO_2_ supported Ir catalysts. (E) Polarization curves of the PEMWEs by using annealed Ir‐Pt‐TiO_2_ catalyst and commercial catalyst.^[^ ^47^ ^]^ Reproduced with permission.^[^ ^47^ ^]^ Copyright 2022, American Chemical Society; Reproduced with permission.^[^ ^56^ ^]^ Copyright 2016, The Royal Society of Chemistry.

There are two most promising and applicable strategies for reducing Ir loading at the OER electrode. One is by mixing or compositing the active IrO_2_ or RuO_2_ component with cheaper transition metal oxides.^[^ ^45^, ^46^, ^47^ ^]^ The key principle of this compositing is to maintain the electronic conductivity of the catalyst while maintaining or improving the mass electrochemical activity of the precious active component due to the highly dispersed nature of the catalytic sites.^[^ ^48^ ^]^ Besides, another attendant merit of this compositing is recognized to be the improved durability, which has been commonly attributed to the inhibited corrosion of the catalysts, slowed active sites loss, or suppressed valence state changes.^[^ ^49^ ^]^ To make this kind of mixing or compositing meaningful, it is critical to increase the threshold limit of the cheaper oxide. IrO_2_/Ta_2_O_5_ with an IrO_2_ content of around 54.2 wt% (70 mol%) is one of the successful examples that gained commercial application at an early stage.^[^ ^50^, ^51^, ^52^ ^]^ However, a further reduction of IrO_2_ content was manifested to be a big barrier, as accompanied by a severe performance degradation. In recent years, many electron‐conductive, corrosion‐resistant, and less expensive oxide supports, such as tin oxide (SnO_2_), titanium oxide (TiO_2_), niobium oxide (Nb_2_O_5_), and so on, have been explored and further modified by fluorine (F)‐doping to enhance the intrinsic electronic conductivity.^[^ ^53^, ^54^, ^55^ ^]^ For example, Datta et al. reported the synthesis of F‐doped SnO_2_ with improved electronic conductivity compared to pristine SnO_2_ to composite with IrO_2_, and a novel single phase [(Sn_0.8_Ir_0.2_)O_2_:F] was generated with the incorporation of F at a certain concentration of 9%–10%.^[^ ^53^ ^]^ The F‐doped (Sn_0.8_Ir_0.2_)O_2_ composite exhibited parent rutile crystal structure with only 20 at% IrO_2_ and comparable OER catalytic activity and durability with pure IrO_2_. The incorporation of F was found to regulate the d‐band center of Ir to render the material essentially mimicking the electronic property and catalytic activity of pure IrO_2_, which could make it cost‐effective for practical PEM water electrolysis as the doping amount of non‐noble metal oxide exceeded a percolation threshold limit of 50 at%, however, the actual performance of this material in PEMWE has not been verified. Regmi et al., recently explored the modification of TiO_2_ support to realize the reduction of IrO_2_ loading through architecture engineering.^[^ ^47^ ^]^ As depicted in Figure 2A, traditional ways of depositing the conductive Ir (or IrO_2_) onto the surface of semiconductor support have found difficult to maintain the electronic conductivity of Ir (or IrO_2_) at low catalyst loadings, according to both the experimental measurements and model predictions (Figure 2B).

Introducing a thin layer of Pt/Au as a conductive conformal layer onto the TiO_2_ nanoparticle was proposed to improve the electrical conductivity of the oxide support, and this supported catalyst architecture was found to render a certain flexibility in reducing the precious metal loading by creating nanostructured layer morphology. An obvious improvement of 141% of the mass activity was observed with the as‐formed Ir‐Pt‐TiO_2_ catalyst (1.02 A mg_Ir_^−1^) compared to the 0.42 A mg_Ir_^−1^ of the commercial catalyst at 1.8 V (Figure 2C), on the basis of a 42% lower precious group metal content (considering the 75 wt% Ir in the commercial catalyst vs. 25 wt% Ir and 18 wt% Pt in Ir‐Pt‐TiO_2_). With the existence of a Pt or Au conductive layer, the TiO_2_ supported Ir catalysts exhibited improved electronic conductivity. Besides, the thermo‐stability of the Pt layer was able to maintain the electronic conductivity of the material after annealing treatment (Figure 2D). Electrolyzer performance curves with and without Ir correction in Figure 2E further prove the maintenance of electrode performance in the case of reducing Ir loading while enhancing the electronic conductivity of the catalyst.

Another promising way to reduce Ir loading at the OER electrode is by metal doping.^[^ ^57^, ^58^, ^59^ ^]^ Although this catalyst modification strategy has some limitations in the doping amount of non‐noble metals, it could bring many other benefits, like improved activity of the catalytic sites with optimized electronic properties and enhanced durability due to a more stable lattice structure.^[^ ^60^, ^61^, ^62^ ^]^

**FIGURE 3:**
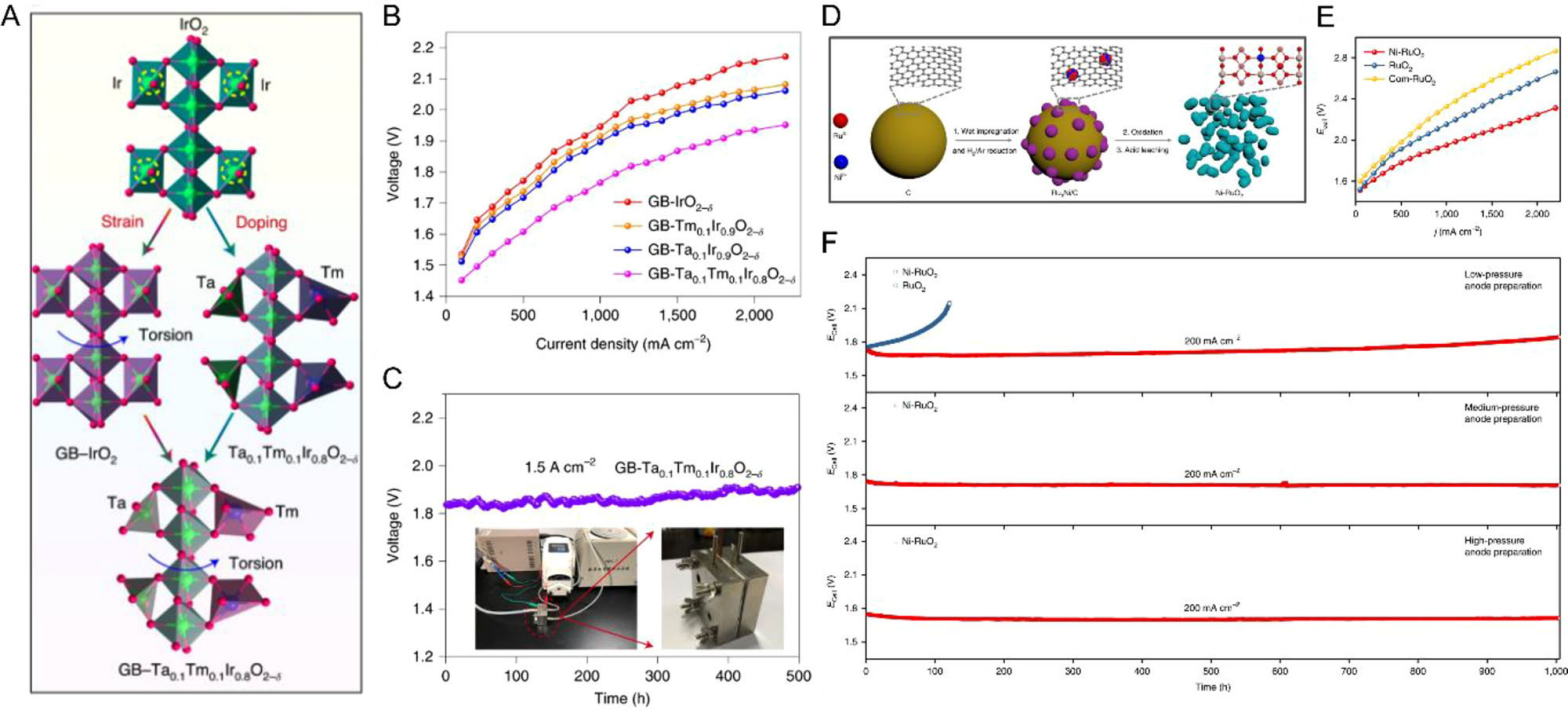
Modification of catalyst electronic properties and lattice structures to enhance activity and stability. (A) Illustration of synergistic grain boundaries, strain, and metals doping effects on enhancing electrochemical activity. (B) Polarization curves of the PEMWEs by applying Ta_0.1_Tm_0.1_Ir_0.8_O_2‐δ_ nanocatalyst and other comparative catalysts in 0.5 m H_2_SO_4_ electrolyte at 50°C. (C) Stability at 1.5 A cm^−2^ of the PEMWE with Ta_0.1_Tm_0.1_Ir_0.8_O_2‐δ_ anode.^[^ ^58^ ^]^ (D) Schematic illustration of the synthesis of Ni‐RuO_2_ catalyst. (E) Polarization curves of the PEMWEs fabricated with the Ni‐RuO_2_ anode and RuO_2_ anodes. (F) Stability of the PEMWEs operated at 200 mA cm^−2^ with the Ni‐RuO_2_ electrodes prepared under different pressures and a comparison with the RuO_2_ electrode.^[^ ^59^ ^]^ Reproduced with permission.^[^ ^58^ ^]^ Copyright 2022, The Author(s), under exclusive license to Springer Nature Limited; Reproduced with permission.^[^ ^59^ ^]^ Copyright 2021, The Author(s), under exclusive license to Springer Nature Limited.

For example, Hao et al., recently reported the development and application of a torsion‐strained Ta_0.1_Tm_0.1_Ir_0.8_O_2‐δ_ nanocatalyst for PEMWE. As illustrated in Figure 3A, by taking advantage of both the metal doping for tuning the metal‐oxygen (M‐O) bonds of the catalytic sites, and the grain boundaries strain to enhance the electrocatalytic activity and possible crystallinity, the PEMWE, by applying the Ta_0.1_Tm_0.1_Ir_0.8_O_2‐δ_ nanomaterial as the anode catalyst at a low mass loading of 0.2 mg cm^−2^ was able to merit over the other commercial Ir catalyst and reported state‐of‐the‐art IrO*_x_* catalysts by delivering an electrolysis current density of 1 A cm^−2^ at 1.766 V (Figure 3B). In addition, the long‐term durability of the PEMWE for over 500 h operation at a high current density of 1.5 A cm^−2^ was proved with the Ta_0.1_Tm_0.1_Ir_0.8_O_2‐δ_ anode, indicating the stability of the crystalline structure to ensure abundant catalytic sites (Figure 3C). Development of Ir‐free RuO_2_‐based anodes for PEMWEs has also been considered as highly meaningful,^[^ ^63^, ^64^ ^]^ because the intrinsic activity of RuO_2_ is higher than that of IrO_2_,^[^ ^65^, ^66^ ^]^ meanwhile the price of Ru (465 USD/Oz) is only one‐tenth of that of Ir (4600 USD/Oz). The long‐term stability of RuO_2_ under acidic OER conditions has long been challenged, which is generally attributed to the over‐oxidation of RuO_2_ to dissolvable higher‐oxidation‐state RuO_4_ species, accompanied by the collapse of catalyst crystal structure during the OER process.^[^ ^67^, ^68^, ^69^ ^]^ To make RuO_2_‐based anodes applicable for practical PEMWE operating conditions, research efforts have now been largely paid on either tuning the Ru oxidation state by introducing electron‐donating elements,^[^ ^70^ ^]^ or regulating the reaction route in a more stable way.^[^ ^68^, ^71^ ^]^ Currently, both approaches are still considered challenging to achieve desirable stability and maintain the superior activity of RuO_2_ itself at the device level. Wu et al., reported the synthesis of Ni‐stabilized RuO_2_ (Figure 3D) with enhanced lattice stability of surface Ru and subsurface oxygen.^[^ ^59^ ^]^ The Ru sites presented a slightly higher oxidation state by Ni doping in Ni‐RuO_2_ at an atomic ratio of 1.4 at% and exhibited a higher catalytic activity for OER compared with the pristine RuO_2_. Electrolyzer performance tests (Figure 3E,F) demonstrated the more favorable activity and stability of Ni‐RuO_2_ as the anode. Although the overall PEMWE efficiency is still behind the industrial level, superior stability with Ni‐RuO_2_ for more than 1000‐h stable operation at 200 mA cm^−2^ was proved, which would be promising for practical applications. In general, from the perspective of material design, strategies have been proposed to reduce the precious metal loading in electrodes. The basic principle in these strategies is to maintain the active site concentration as much as possible, while avoiding sacrificing the stability of the bulk material through appropriate compositing or doping.

### 2.2 Strategies for improving catalyst utilization in PEMWEs

At present, the generally used Ir loading in commercialized PEMWEs is at a level of 1–2 mg cm^−2^.^[^ ^72^ ^]^ Such a high catalyst loading, on the one hand, is to compensate for the low catalytic sites utilization within the catalyst layer due to the insufficient electronic conductivity,^[^ ^73^, ^74^ ^]^ on the other hand, it is to maintain the long‐term stability of the electrode performance.^[^ ^75^, ^76^ ^]^ For the PEMWE electrode in the form of CCM, the catalyst layer is very thin, so the ideal distribution of catalyst particles and ionomers has been a long‐standing elusiveness in fabricating the most‐effective electrode for PEMWE, which attracts tremendous interest from researchers to explore the balance between the electron conductivity and the ionic conductivity within the catalyst layers.^[^ ^77^ ^]^

**FIGURE 4:**
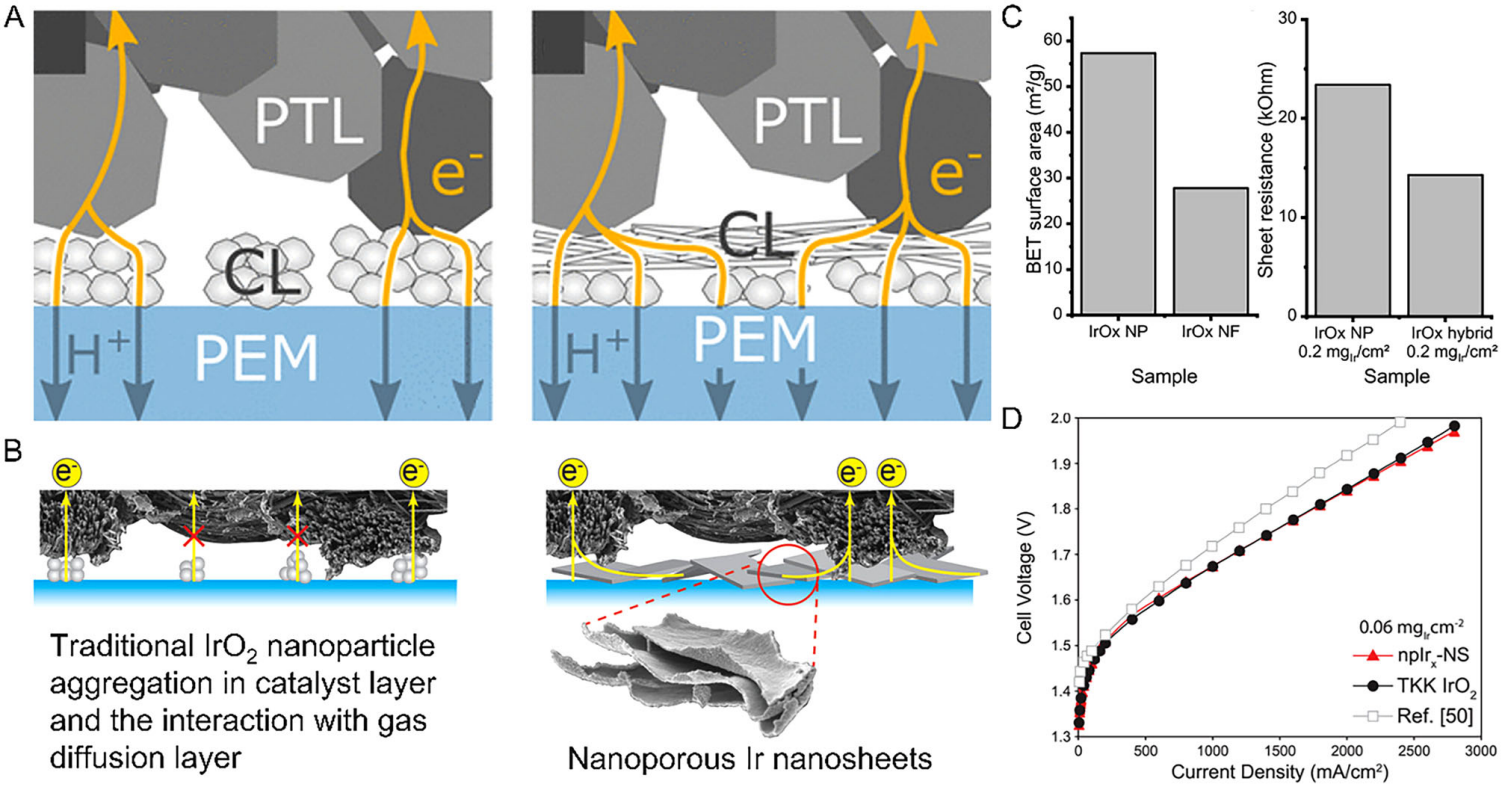
Electron conductance optimization within the catalyst layer (CL) and at the interface between the CL and the porous diffusion layer (PTL). (A) Schematic illustration of the anode catalyst layers with traditional IrO*_x_* nanoparticles and the combination of IrO*_x_* nanofibers. (B) BET surface area of the IrO*_x_* nanoparticles and the IrO*_x_* nanofibers and the sheet resistance of the as‐formed catalyst layers.^[^ ^78^ ^]^ (C) Schematic illustration of the anode catalyst layers as‐formed with IrO_2_ nanoparticle aggregates, and with nano porous Ir nanosheets. (D) PEMWEs performance with TKK IrO_2_ catalyst (0.17 mg_Ir_ cm^−2^) and Ir nanosheets at an ultra‐low Ir loading of 0.06 mg_Ir_ cm^−2^, compared to commercial CCM.^[^ ^79^ ^]^ Reproduced with permission.^[^ ^78^ ^]^ Copyright 2020, American Chemical Society; Reproduced with permission.^[^ ^79^ ^]^ Copyright 2021, Wiley‐VCH GmbH.

The construction of uniform catalyst layers has been demonstrated in some works by exploiting unique catalyst morphology to build continuous electronic conductance and ionic conductance pathways.^[^ ^78^, ^79^ ^]^ For example, Hegge et al., reported the combination of IrO*_x_* nanofibers with traditional IrO*_x_* nanoparticles to form the anode catalyst layer (Figure 4A) and Chatterjee et al., reported the application of nanoporous Ir nanosheets to form the anode catalyst layer (Figure 4B).^[^ ^79^ ^]^ For catalyst layers composed of oxide catalysts, the insufficient electronic conductivity is considered the main barrier to catalytic site utilization, especially when reducing the catalyst loadings.^[^ ^80^ ^]^ Both works aim to improve the electronic conductivity during the electrode reaction in PEMWEs, due to catalyst agglomeration, uneven ionomer dispersion, and a limited contact interface between the catalyst layer and porous diffusion layer. The 1D IrO*_x_* nanofiber though exhibited lower electrode surface area compared with IrO*_x_* nanoparticle, the in‐plane electronic conductivity with IrO*_x_* nanofiber was greatly improved at the same catalyst loading (Figure 4C). Besides, benefiting from the improved mass transportation brought by the nanoporous structure of Ir nanosheets, and the reduced ohmic resistance of the improved lateral connectivity and interconnected electron conductance, an ultra‐low Ir loading of 0.06 mg cm^−2^ was achieved without sacrificing electrode performance (Figure 4D).

**FIGURE 5:**
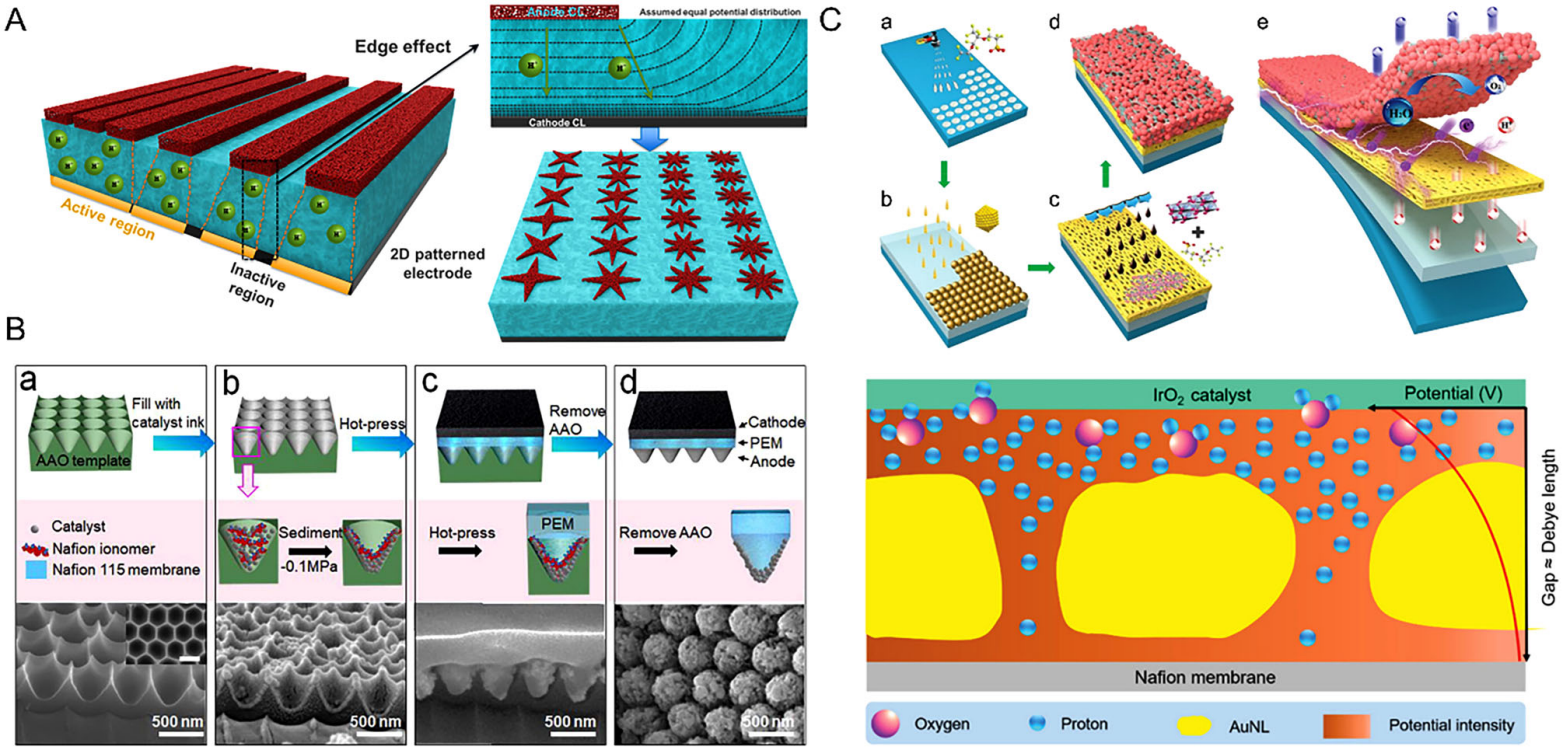
Interface regulation strategies between the PEM and the catalyst layer. (A) Schematic illustration of the 2D‐patterned electrodes with edge effect.^[^ ^81^ ^]^ (B) Fabrication procedures of the gradient ordered anode and the electrode surface morphologies.^[^ ^82^ ^]^ (C) Construction of the IrO_2_ anode with Au mesoporous nanolayer and schematic illustration of the transport behaviors at the interface.^[^ ^83^ ^]^ Reproduced with permission.^[^ ^81^ ^]^ Copyright 2022, American Chemical Society; Reproduced with permission.^[^ ^82^ ^]^ Copyright 2022, American Chemical Society; Reproduced with permission.^[^ ^83^ ^]^ Copyright 2020, Wiley‐Vch Verlag GmbH & Co. KGaA, Weinheim.

Although uniform catalyst layers are commonly used for PEMWEs, some researchers recently also reported the fabrication of discontinuous catalyst layers that featured 2D‐patterned structures (Figure 5A).^[^ ^81^ ^]^ An edge effect with the patterned electrode was proposed to mainly influence the internal potential distribution and proton conduction pathway within the membrane. As a result, the actual‐effective electrode area, though proportional to the physical anode area, could be larger than it is. Therefore, with the optimized catalyst strip and gap sizes, an ideal electrode performance was achieved by saving 61% of the precious catalyst (IrO_2_) loading within the anode but did not impair the electrode performance with the enhanced catalyst mass activity. The non‐uniform catalyst layer as an emerging electrode structure has sparked widespread interest among researchers.^[^ ^82^, ^83^ ^]^ Dong et al., reported the design of a gradient ordered catalyst layer with the purpose of optimizing the contact interface between the catalyst layer and the PEM.^[^ ^82^ ^]^ The electrode fabrication process is illustrated in Figure 5B. An extended catalyst layer and membrane interface were formed after the template filling and hot‐pressing, as a consequence, the electrochemical active area of the electrode was increased by 4.2 times through this electrode fabrication strategy, greatly enhancing the catalytic site utilization. The PEMWE with the gradient ordered anode (Ir loading of 0.2 mg cm^−2^) exhibited a decreased ohmic overpotential of 8.7% and a decreased mass transportation overpotential of 13.9%, compared to that with the conventional anode (Ir loading of 2 mg cm^−2^), while presenting almost the same efficiency and durability.

The interface regulation between the catalyst layer and the membrane could also be realized by introducing a functional sublayer. The sublayer with a high electronic conductivity, under the premise of not impeding ion conduction, would greatly improve the mass activity of the catalyst.^[^ ^83^, ^84^ ^]^ For example, Yang et al., reported the build of Au sublayers with different thicknesses for providing both the electron and the proton nano highways to improve the catalytic sites utilization of the electrode.^[^ ^83^ ^]^ The fabrication process is illustrated in Figure 5C with the schematic diagram of one as‐formed Au nanolayer with straight mesopores. In general, the electric potential profile within this layer should be able to provide sufficient in‐plane electronic conductivity while also facilitating through‐plane proton transport during the electrode reaction. Thus, the thickness of this nanolayer and the Au particle size should be precisely controlled. Concretely, a thin nanolayer with ultra‐fine Au nanoparticles could be beneficial for driving the protons through, while the low local potential could be insufficient to facilitate effective electron conduction.^[^ ^85^ ^]^ However, a thick nanolayer with larger Au nanoparticles also features deeper mesopore channels (a larger gap), which could uniformly utilize the catalytic sites, but may also hinder the migration of protons.

### 2.3 Local electrode‐reaction‐environment regulation in PEMWEs

**FIGURE 6:**
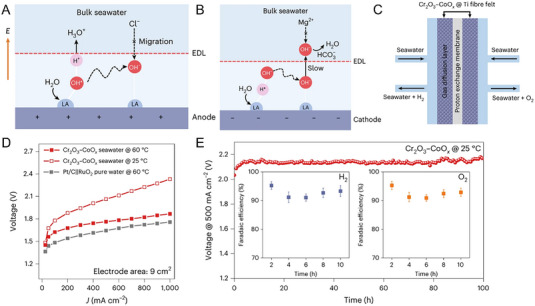
Mechanism for local microenvironment adjustment on the (A) Cr_2_O_3_–CoO*_x_* OER electrode, (B) Cr_2_O_3_–CoO*_x_* HER electrode. (C) Schematic illustration of the Cr_2_O_3_–CoO*_x_* symmetrical seawater PEMWE with the Cr_2_O_3_–CoO*_x_* as both the anode and cathode catalyst under seawater feeding. (D) A comparison of polarization curves of the Cr_2_O_3_–CoO*_x_* symmetrical seawater electrolyzers and the state‐of‐the‐art Pt/C‖RuO_2_ PEMWE. (E) Stability of the Cr_2_O_3_–CoO*_x_* symmetrical seawater electrolyzer operated at 500 mA cm^−2^, with the inset showing the Faradaic efficiencies of H_2_ and O_2_ production. Reproduced with permission.^[^ ^12^ ^]^ Copyright 2023, The Author(s), under exclusive license to Springer Nature Limited.

Direct seawater electrolysis through PEMWE technology is highly attractive. From one perspective, the electric efficiency for pure hydrogen production is the highest with PEMWE among several low‐temperature water electrolysis technologies.^[^ ^86^, ^87^ ^]^ From another perspective, it will eliminate the cost of seawater desalination and purification before supplying it to PEMWE and reduce the system volume.^[^ ^88^ ^]^ Guo et al., recently demonstrated a direct seawater electrolysis approach in PEMWE by adjusting the local reaction environment.^[^ ^12^ ^]^ A preferential OH^−^‐enriched microenvironment on the catalyst surface was created by constructing a hard Lewis acid layer (Cr_2_O_3_) over the CoO*_x_* catalyst. During the OER process, water molecules split, leaving the surface covered with large amounts of OH^*^, which were promoted to form OH^−^ due to the electrical double layer (EDL) near the catalyst surface (Figure 6A). In this case, chlorine oxidation was inhibited by hindering the approach of chloride ions to the catalyst surface. While on the negatively charged cathode surface with Cr_2_O_3_–CoO*_x_*, the HER activity and stability were boosted due to the restriction of OH^−^ on the Cr_2_O_3_ layer within the EDL to maintain the reaction pH and prevent precipitation (Figure 6B). A symmetrical electrolyzer using this Cr_2_O_3_–CoO*_x_* catalyst for both the OER and HER electrodes, with both sides feeding with filtered natural seawater without acidification or alkalization process, was able to deliver a comparable performance to the state‐of‐the‐art PEMWE with RuO_2_ anode and Pt/C cathode with pure water feeding (Figure 6C,D). Notably, electrolysis durability in the PEMWE for 100‐h operation at 500 mA cm^−2^ was achieved with the Faradaic efficiencies of ∼93% and ∼92% for H_2_ and O_2_ production (Figure 6E).

## 3 ELECTRODE ENGINEERING TOWARD AEMWES

AEMWE started to attract attention since 2012,^[^ ^89^ ^]^ is now still considered an emerging technology at the research and development stage.^[^ ^90^ ^]^ The most attractive merit of AEMWE is that the alkaline operating condition expanded the choices of catalysts for OER and HER electrodes, making a variety of non‐noble metal oxides applicable, for example, perovskites, spinels, and layered double hydroxides (LDH).^[^ ^91^, ^92^, ^93^, ^94^ ^]^ Different from traditional alkaline electrolysis cells, AEMWE is able to achieve comparable electrolysis efficiency to PEMWE, as advanced AEMs have achieved high hydroxide conductivities of higher than 200 mS cm^−1^, with a thickness of less than 50 μm.^[^ ^95^, ^96^ ^]^ However, the chemical stability of the AEMs still remains challengeable for large‐scale application in the AEMWE, as the electron‐deficient cationic moieties appended to the polymer backbones for maintaining conductivity are vulnerable to attack by hydroxide.^[^ ^97^ ^]^ To take advantage of the alkaline condition, electrode engineering at AEMWE focuses more on the development of 3D porous electrodes,^[^ ^98^, ^99^ ^]^ based on the CCS method. Especially for the metal‐based self‐supporting porous electrodes, which greatly increase the active surface area, by allowing the distribution of more active sites while ensuring continuous electronic conductivity compared to the CCM electrodes. Herein, we present an overview of the recent progress on constructing 3D porous self‐supporting electrodes with optimized catalyst distribution, building an effective ionic conductance pathway, as well as designing energy‐saving ways toward seawater electrolysis.

### 3.1 3D metal‐based self‐supporting porous electrodes for AEMWEs

**FIGURE 7:**
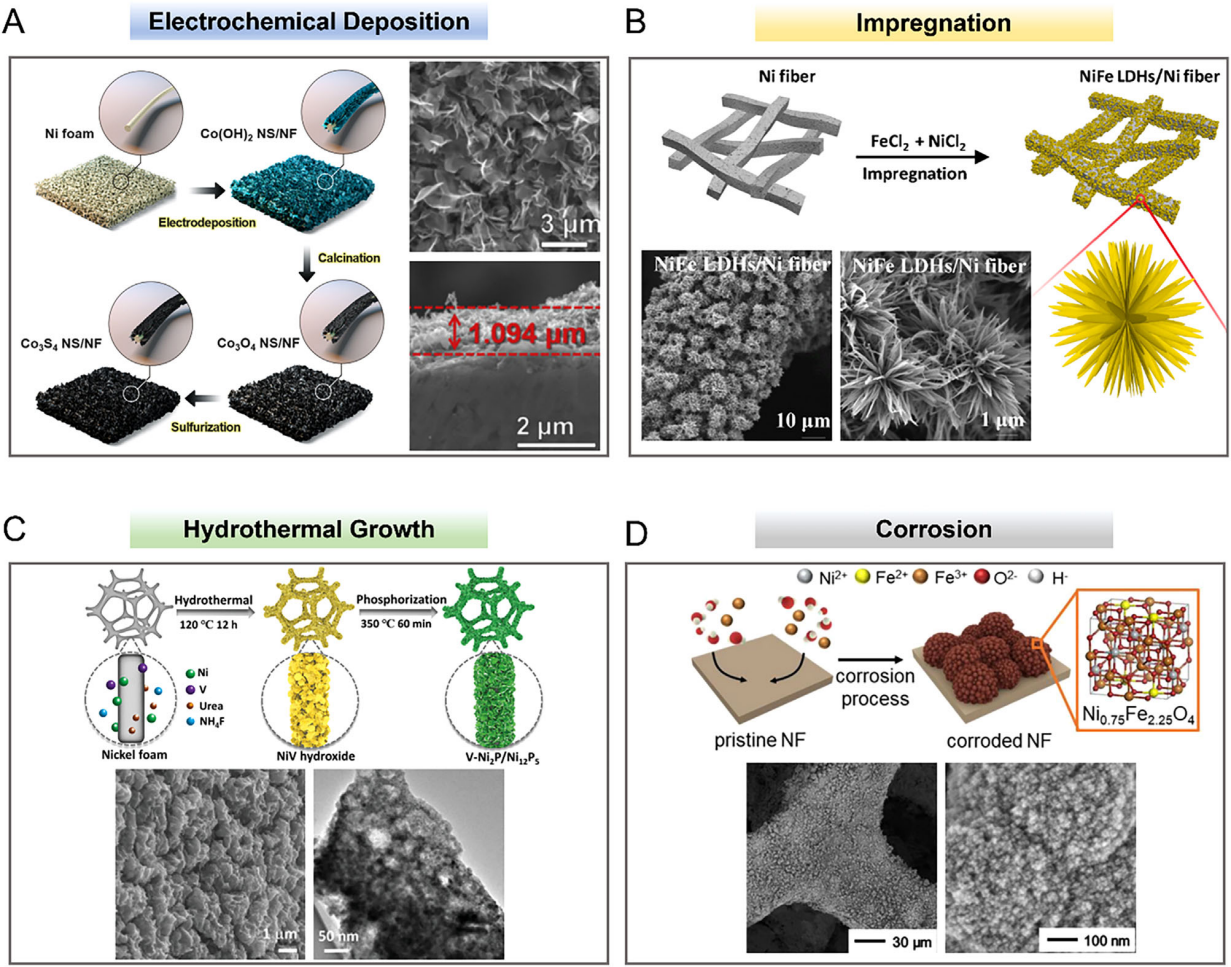
Ni foam or Ni fiber felt‐based electrodes prepared by various strategies and the electrode morphologies characterized by scanning electron microscopy (SEM). (A) The Co_3_S_4_ NS/NF electrode prepared by electrochemical deposition.^[^ ^112^ ^]^ (B) The NiFe LDHs/Ni fiber electrode prepared by impregnation,^[^ ^110^ ^]^ (C) The V‐Ni_2_P/Ni_12_P_5_ electrode prepared by hydrothermal growth^[^ ^111^ ^]^ and (D) the Ni_0.75_Fe_2.25_O_4_ electrode prepared by corrosion method.^[^ ^107^ ^]^ Reproduced with permission.^[^ ^112^ ^]^ Copyright 2019, Hydrogen Energy Publications LLC. Published by Elsevier Ltd.; Reproduced under the terms of the CC‐BY license.^[^ ^110^ ^]^ Copyright 2022, The Author(s); Reproduced with permission.^[^ ^111^ ^]^ Copyright 2022, Wiley‐VCH GmbH; Reproduced with permission.^[^ ^107^ ^]^ Copyright 2020, Elsevier B.V.

The mild alkaline environment within AEMWEs makes 3D metal foams, with their cheap price and mature manufacturing process, the best electrode candidates.^[^ ^1^ ^]^ The most commonly used metal foams at present in AEMWEs are the Ni foam and the Cu foam.^[^ ^100^, ^101^ ^]^ Besides the intrinsic catalytic ability of these two materials under alkaline media, other properties of these two foams, like rich porous‐structure, simple preparation technique, and superior electronic conductivity, are all highly desirable as electrode substrates.^[^ ^98^, ^102^ ^]^ The commonly used preparation and fabrication methods for metal foam‐based electrodes used in AEMWEs mainly include electrochemical deposition,^[^ ^103^ ^]^ in situ hydrothermal growth,^[^ ^104^, ^105^ ^]^ impregnation,^[^ ^106^ ^]^ corrosion engineering,^[^ ^107^ ^]^ and ultrasonic spraying,^[^ ^108^, ^109^ ^]^ etc. Figure 7 shows the representative electrode surface morphologies formed via different preparation techniques in the current literature.^[^ ^107^, ^110^, ^111^, ^112^ ^]^ It can be found that with the existence of metal foams as supports, the electrode surfaces as‐obtained mostly present nanorods, nanoarrays, or nanosheets structures by controlling the deposition and growth of the electrocatalysts. This kind of electrode surface resulted in a desirable electrode reaction interface with highly exposed active surface area and abundant hierarchical pores, so as to ensure sufficient active sites and mass transportation, especially for high‐current water electrolysis in the electrolyzer.

For metal foam‐based self‐supporting porous electrodes, the construction of effective ion (anion) conduction pathways within the electrode is also a significant concern for achieving favorable electrolyzer performance.^[^ ^121^ ^]^ Referring to the operating conditions of AEMWEs as listed in Table 1, alkaline solution (0.1–6 m KOH) is generally used to support the electrode reaction on 3D self‐supporting porous electrodes. In contrast to the commonly used CCM electrodes in PEMWEs, metal foam‐based self‐supporting porous electrodes, on the one hand, avoid the weak contact interface between the porous diffusion layer and the catalyst layer, thus enabling efficient electron conduction during the reaction and improving the catalytic sites utilization. On the other hand, self‐supporting electrode assembly enables millimeter‐thick electrodes with rich pores to provide sufficient accessible active sites for the electrode reaction. Alkaline solution is therefore used to enable the effective ion conduction to the active center, while also maintaining the high OH^−^ conductivity of the membrane.

**TABLE 1:** A summary of representative 3D metal‐based self‐supporting porous electrodes developed for AEMWEs: Preparation strategy, morphology, application, and electrolyzer performance.

Electrode		Morphology	Preparation strategy	Reaction	Electrolyzer performance with test conditions	Ref.
Substrate	Catalyst					
Ni foam	Fe_0.2_Ni_0.8_‐P_0.5_S_0.5_	Nano‐island arrays	Electrodeposition	HER OER	1.5 A cm^−2^ at 1.8 V 60°C, 1 m KOH	[103]
Ni foam	Cu_0.81_Co_2.19_O_4_	Nanosheets	Electrodeposition	OER	431 mA cm^−2^ at 2.0 V 45–48°C, 1 m KOH	[112]
Ni foam	Co_3_S_4_	Nanosheets		HER		
Ni foam	Ni_2_P–Fe	Nanoparticles	Impregnation	OER	1 A cm^−2^ at 1.73 V 80°C, 1 m KOH	[106]
Ni fiber	NiFe LDHs	Nanoflower	Impregnation	OER	0.5 A cm^−2^ at 1.68 V 70°C, 1 m KOH	[110]
Cu foam	Cu(OH)_2_@NiFe LDHs	Nanorod arrays	Impregnation	OER	0.5 A cm^−2^ at 1.56 V 70°C, 1 m KOH	[113]
Ni foam	Ni_0.75_Fe_2.25_O_4_	Nanoparticles	Corrosion	OER	2.0 A cm^−2^ at 1.9 V 42–45°C, 1 m KOH	[107]
Ni foam	Ni‐doped FeOOH	Nanosheets	Corrosion	OER	729 mA cm^−2^ at 1.70 V 50°C 1.0 m KOH + Seawater	[114]
Ni foam	NiFeV LDHs	Nanosheets	Corrosion	OER	2108 mA cm^−2^ at 1.8 V 50°C, 1 m KOH	[115]
Ni foam	CuCo_2_O_4_	Chestnut burrs	Hydrothermal	OER	1.4 A cm^−2^ at 1.9 V 45°C, 1 m KOH	[104]
Ni foam	Ir‐Ni/Mo_5_N_6_	Nanowire arrays	Hydrothermal	HER OER	2.1 A cm^−2^ at 2.0 V 80°C, 1 m KOH	[116]
Ni foam	Ni_2_P/Ni_7_S_6_	Nanosheets	Molten salt	OER	1000 mA cm^−2^ at 1.88 V 75°C, 1 m KOH	[117]
Ni foam	MoNiO_4_	Nanorod arrays	Impregnation	HER	0.55 A cm^−2^ at 2 V 60°C, 1 m KOH	[118]
Pt‐coated Ni foam	Ni‐IrO_x_	Nanoparticle agglomerates	Dip coating	OER	1454.8 mA cm^−2^ at 1.8 V 70°C, 1 m KOH	[119]
Ni foam	La_0.5_Sr_0.5_CoO_2.91_	Nanoparticle agglomerates	Spray coating	OER	1000 mA cm^−2^ at ∼1.75 V 70°C, 0.1 m KOH	[109]
Ni foam	LaSr_2.7_Co_1.5_Fe_1.5_O_10_	Nanoparticles	Spray coating	OER	2.01 A cm^−2^ at 2.00 V 60°C, 6 m KOH	[108]
Ni foam	NiFe_2_O_4_	Clusters	Spray coating	OER	2.0 A cm^−2^ at 2.0 V 60°C, 1 m KOH	[120]

Abbreviation: LDHs, layered double hydroxides.

**FIGURE 8:**
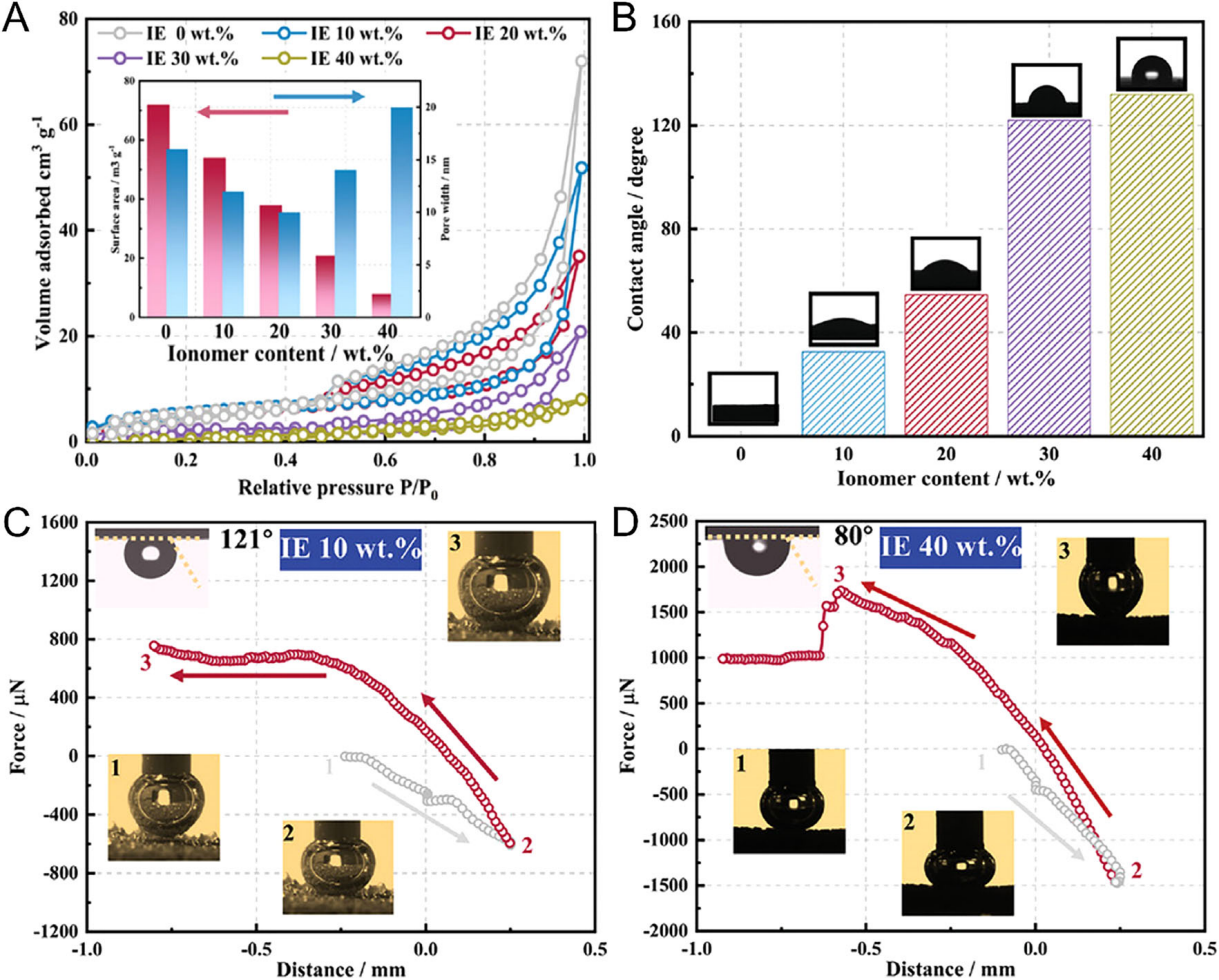
Ni foam‐based self‐supporting electrode porous structure and surface properties changes with different ionomer coverage. (A) N_2_ adsorption–desorption isotherms and Brunauer–Emmett–Teller (BET) specific surface areas, and (B) water contact angles of the FeNi LDH integrated electrodes with the ionomer content from 0 to 40 wt%. Gas bubble adhesions of the integrated electrodes with (C) 10 wt% and (D) 40 wt% ionomer content.^[^ ^122^ ^]^ Reproduced with permission.^[^ ^122^ ^]^ Copyright 2022, Wiley‐VCH GmbH.

In order to achieve pure water electrolysis in AEMWEs, the use of basic ionomer to construct an efficient ion‐conducting network has also been investigated. Except for the electrodes prepared by the spray coating or dip coating method, in which catalysts are pre‐mixed with ionomer first to form the uniform catalyst slurry, in the other electrode preparation strategies, catalysts are mostly arranged onto the porous supports by in situ growth first, so the fabrication of self‐supporting electrodes for AEMWEs generally involves a “two‐step” procedure followed by an ionomer network construction.^[^ ^122^ ^]^ Wan et al., recently investigated the arrangement of an ionomer network by ultrasonic spraying onto an integrated FeNi‐based electrode with vertically aligned LDHs on the surface.^[^ ^122^ ^]^ Rational distribution of the ionomer plays a significant role in improving catalyst utilization by providing effective ion transport pathways during the electrode reaction, especially for metal foam‐based electrodes with a relatively large thickness. Besides the ion conduction, it was found that the ionomer covering on the electrode surface would also affect the porous structure and the surface wettability/hydrophobicity, which are directly related to the gas/liquid mass transportation at the reaction interface.^[^ ^123^ ^]^ A fine regulation of the ionomer content from 0 to 40 wt% was found to gradually occupy the voids (macropores) in the electrode, leading to a decreased BET specific surface area (Figure 8A), and the contact angle became larger with the increased ionomer content (Figure 8B), indicating a more hydrophobic electrode surface as formed. Noticeably, an over‐hydrophobic electrode surface with the ionomer content of higher than 30 wt% was believed to bring a negative effect to the electrode reaction due to the impeded reactant penetration and the decreased exposure of the active sites. Concomitantly, a decrease in the electrode surface roughness by increasing the ionomer content was found to show enhanced bubble adhesion capability (Figure 8C,D), which could be detrimental to the timely release of the generated gas bubbles during the electrode reaction.

Besides metal foams, other supports have also been explored for fabricating the 3D self‐supporting electrodes, as the mild alkaline condition in AEMWE provides certain flexibility in electrode material selection. Park et al., recently reported the fabrication of a series of unified electrodes via electrodeposition based on different substrates, including Ti paper, stainless steel paper, and Ni foam, to investigate the influence of electrode fabrication parameters to the AEMWE efficiency and stability.^[^ ^124^, ^125^ ^]^ The self‐supporting electrodes as‐prepared on the basis of various supports exhibited a performance trend of Ni foam < Ti paper < stainless steel paper in AEMWEs. According to the electrochemical impedance spectroscopy (EIS) measurements, the obvious performance difference in AEMWEs was mainly from the ohmic resistance, however, it is worth noting that the difference lies in the ohmic resistance of the electrolyzers may also come from the changed cell assembly states when fabricating with electrodes of different thickness.^[^ ^126^ ^]^

### 3.2 Electrode design toward cost‐effective hybrid water electrolysis

The sluggish kinetics of the anodic OER determines that the energy consumption of conventional water electrolyzers is at a high level of 4.8–5.5 kWh Nm^−3^ (H_2_) corresponding to the applied voltage in the range of 1.8–2.0 V. Hybrid water electrolysis has been proposed in recent years by replacing the anodic OER with an “easier” oxidation reaction to reduce the overall potential for water electrolysis.^[^ ^127^ ^]^ Advances in exploring the alternative energy‐saving oxidation reactions mainly involve alkaline environment, because the utilization of hydroxyl groups (OH^−^) to react with organic molecules provides more possibilities for this kind of hybrid water electrolysis. According to different sources of the organic matter, the alternative anodic oxidation reactions can be classified into three categories: the reactions with the existence of organic molecules as sacrificing agents,^[^ ^128^, ^129^, ^130^ ^]^ the reactions for generating value‐added products,^[^ ^131^, ^132^, ^133^ ^]^ as well as the reactions involving the depletion of environmental pollutants.^[^ ^134^, ^135^ ^]^

**FIGURE 9:**
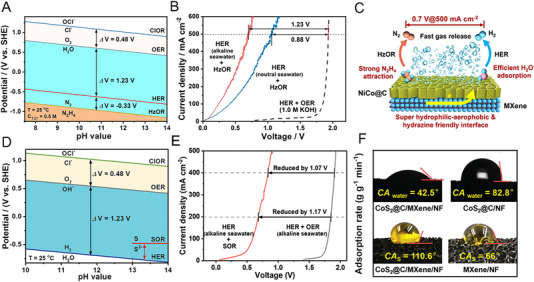
Hybrid electrolyzer design to enable cost‐effective water electrolysis. (A) Pourbaix diagram of the HzOR, HER, OER, and ClOR in neutral to alkaline seawater. (B) An electrolysis performance comparison of the hybrid electrolyzers (HER + HzOR) under neutral and alkaline seawater conditions and the water electrolyzer (HER + OER). (C) Schematic illustration of the merits of the NiCo@C/MXene/CF electrode.^[^ ^13^ ^]^ (D) Pourbaix diagram of the SOR, HER, OER, and ClOR under alkaline conditions. (E) An electrolysis performance comparison of the hybrid electrolyzer (HER + SOR) and the water electrolyzer (HER + OER). (F) Optimized hydrophilic and sulfur‐phobic properties of the CoS_2_@C/MXene/NF electrode with decreased water contact angle (CA_water_) and increased sulfur contact angle (CA_s_).^[^ ^14^ ^]^ Reproduced under the terms of the CC‐BY license.^[^ ^13^ ^]^ Copyright 2021, The Author(s); Reproduced with permission.^[^ ^14^ ^]^ Copyright 2022, Wiley‐VCH GmbH.

Research works related to the development of catalysts for these three categories of oxidation reactions emerged prosperously in the past 10 years and are summarized in a recent review by Du et al.,^[^ ^127^ ^]^ so here we mainly focus on the development of electrodes toward the application in membrane‐based electrolyzers. The application of ion exchange membrane provides an extra benefit in separating the reaction environment of the cathode from the anode, thus enabling the production of high‐purity hydrogen. By taking advantage of the AEMWE technology and the hydrazine oxidation reaction (HzOR), Sun et al. recently reported an energy‐saving hydrogen production strategy by seawater electrolysis.^[^ ^13^ ^]^ One the one hand, the oxidation potential of the HzOR (−0.33 V vs. RHE) is much lower than the OER (1.23 V vs. RHE) and the chlorine oxidation reaction (ClOR, 1.71 V vs. RHE) as shown in the Pourbaix diagram (Figure 9A). Thus, seawater can be directed used for the HER at the cathode, and even chloride ions exchanged to the anode via AEM can be avoided from oxidation to toxic and corrosive chlorine species such as Cl_2_, and ClO^−^. On the other hand, the hybrid seawater electrolyzer (with the overall performance shown in Figure 9B) achieved the hydrogen production under industrial‐scale current densities at a dramatically lower energy consumption of only 2.75 kWh Nm^−3^ (H_2_). A self‐supported electrode was fabricated by assembling the mesoporous NiCo‐decorated carbon nanosheets (NiCo@C) onto the MXene‐wrapped Cu foam, which was applied as both the cathode for the HER and the anode for the HzOR. As illustrated in Figure 9C, besides the strong chemical interaction exhibited on the NiCo sites with the N_2_H_4_ molecules, which contributed to the intrinsically high activity of the NiCo sites for the HzOR, the favorable electrode performance was also attributed to the desirable interfacial properties as‐brought by the MXene layer with superior electronic conductivity, super aerophobic‐hydrophilic surface environment, and N_2_H_4_‐friendly interface. The successful implementation of HzOR in real AEMWE could also bring confidence in applying the novel catalysts for HzOR under alkaline environment as proposed in recent years.^[^ ^136^ ^]^ Another example for the successful application of hybrid water electrolysis in a membrane electrolyzer was reported by Zhang et. al.,^[^ ^14^ ^]^ by combining the sulfion oxidation reaction (SOR, −0.48 V vs. RHE) at the anode and the HER at the cathode (Figure 9D), an energy‐saving hydrogen yield was achieved at 2.32 kWh Nm^−3^ (H_2_) by cutting down the electrolysis voltage to 0.97 V at an electrolysis current of 300 mA cm^−2^ (Figure 9E). Similarly, MXene‐wrapped Ni foam with macroporous feature was used to fabricate the self‐supporting electrodes. CoS_2_ and CoO nanosheets which decorated with amorphous carbon were uniformly assembled onto the 3D MXene‐wrapped Ni foam scaffold to serve as the anode for the SOR and the cathode for the HER, respectively. The considerable hybrid electrolyzer performance was attributed to the integrated advantages of the electrode. Besides the sufficient active sites enabled by the macroporous scaffold and mesoporous catalyst nanosheets, the electrode surface was featured with optimized hydrophilic and sulfur‐phobic properties (Figure 9F), which on the one hand, alleviated the blockage of the pores within the electrode because of the generation of solid sulfur during the SOR, meanwhile, enhanced the wettability of the electrode for benefiting the HER and the SOR.

## 4 ADVANCED MEMBRANE‐BASED WATER ELECTROLYZER TECHNOLOGIES

At the current stage, green hydrogen production through both the low‐temperature PEMWE and AEMWE technologies cannot ignore a key limitation for long‐term operation by employing impure water as feedstock, due to the influence of impurity ions crossover to the longevity of the membranes.^[^ ^137^, ^138^ ^]^ As pure water is not universally available and needs to be sourced from ground water, city water, lake or sea water, and so on, through complex water treatment and purification, the high capital cost of green hydrogen production associated with this part is hard to be reduced.^[^ ^139^ ^]^ Direct seawater electrolysis has recently become increasingly attractive, which could possibly provide an ultimate solution to this issue, as seawater makes up >96% of the water source on earth.

**FIGURE 10:**
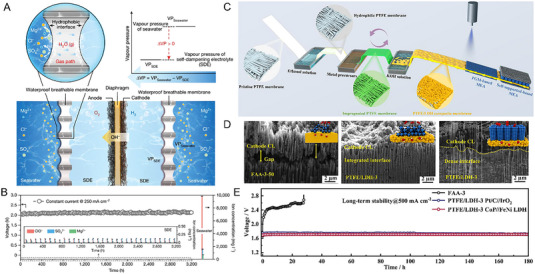
PTFE membrane‐based advanced water electrolysis technologies. (A) The concept of applying PTFE membrane for direct seawater electrolysis via a spontaneous liquid–vapor–liquid migration mechanism. (B) The 3200‐h durability test for the scaled‐up seawater electrolysis system at 250 mA cm^−2^, and the representative ions concentration in the electrolyte.^[^ ^15^ ^]^ (C) A large‐scale MEA fabrication process based on the modified PTFE membrane by pore‐filling strategy. (D) The cross‐section images of the MEAs as‐formed based on the commercial FAA‐3−50 membrane and the PTFE/LDH composite membrane with integrated interfaces. (E) The durability tests for the alkaline electrolysis systems at 500 mA cm^−2^ with the PTFE/LDH composite membrane and the commercial FAA‐3−50 membrane.^[^ ^141^ ^]^ Reproduced with permission.^[^ ^15^ ^]^ Copyright 2022, The Author(s), under exclusive licence to Springer Nature Limited; Reproduced with permission.^[^ ^141^ ^]^ Copyright 2021, Elsevier B.V.

Researchers have been seeking feasible ways for direct seawater electrolysis by applying durable seawater membranes. For example, Xie et al., recently reported the combination of a porous PTFE‐based membrane to achieve large‐scale and stable direct seawater electrolysis.^[^ ^15^ ^]^ The PTFE membrane is intrinsically a robust superhydrophobic microporous fibrous substance that has strong corrosion resistance to seawater and possesses self‐cleaning ability.^[^ ^140^ ^]^ What makes this membrane‐based electrolyzer merit over traditional alkaline electrolysis for direct seawater electrolysis is that the gas‐path interface within the membrane enables the self‐dampening of the concentrated KOH electrolyte with biased diffusion of water vapor from seawater, while preventing liquid solution from entering its pores, as well as intrinsically blocking any dissolved ions (ClO^−^, SO_4_^2−^ and Mg^2+^) in seawater (as illustrated in Figure 10A). Besides, the self‐cleaning capability of the PTFE membrane due to the strong repulsion of water droplets also protects the surface from the attachment of microorganisms in seawater. As a consequence, the scaled‐up PTFE membrane‐based direct seawater electrolysis system (3,696 cm^2^) was proved to survive an extraordinary long‐term operation at 250 mA cm^−2^ for over 3200 h at an attractively low energy consumption of only 5.0 kWh Nm^−3^ H_2_ without any performance or component failure (Figure 10B).

A green and scalable pore‐filling technology has been proposed recently to modify PTFE membrane with satisfying hydroxide conductivity, excellent wettability, and exceptional alkaline stability for advanced alkaline water electrolysis.^[^ ^141^ ^]^ As illustrated in Figure 10C, a large‐scale MEA fabrication process was proposed with several procedures. First, by going through a series of surface modification, impregnation and controlled LDH formation, the PTFE/LDH composite membrane was obtained at a certain degree of pore‐filling with LDH, which has been proven with fine hydroxide ion conductivity.^[^ ^142^, ^143^ ^]^ Then, precious‐metal‐based catalyst layer was deposited onto the membrane to form a CCM, or precious‐metal‐free Ni‐foam based self‐supporting electrodes were applied to finally form the MEA. Both of the electrode fabrication strategies were proved to be available due to the modified PTFE membrane surface properties. The PTFE/LDH composite membrane was featured with a rough and modified hydrophilic surface, was thus able to form an integrated interface with the catalyst layer (Figure 10D). Compared to that with the traditional FAA‐3−50 AEM with a flat surface, the electrolyzer by applying this PTFE/LDH composite membrane‐based MEA exhibited much lower ohmic resistance, and achieved a high electrolysis current density of 1.0 A cm^−2^ at only 1.8 V (60°C, 1 m KOH). Besides, the electrolysis durability based on the PTFE membrane was also demonstrated in this work, with the electrolyzer stably operated at 500 mA cm^−2^ for over 180 h (Figure 10E).

## 5 CONCLUSION AND PERSPECTIVES

In this review, we aim to provide a comprehensive overview of the advanced electrode engineering toward the most promising membrane electrolyzer technologies, and get access to the wide interests of the research community in promoting the fundamental catalyst/electrode development to practical application in water electrolyzers for green hydrogen production. Electrode engineering strategies customized for different electrolyzers, including the most representative polymer electrolyte membrane electrolyzers (PEMWEs and AEMWEs) and the advanced water electrolyzers by applying PTFE membrane, are thoroughly discussed. The main conclusions are as follows:

1) For the compacted CCM‐based electrodes commonly used in PEMWEs, in order to achieve sufficient catalyst utilization and reduce precious metal loading within the catalyst layer, one of the feasible ways by reducing the most active IrO_2_ or RuO_2_ components is via non‐precious metal‐doping or metal‐oxide‐support compositing. Strategies have been developed to maintain the electronic conductivity of the catalyst required for forming the catalyst layer, for example, oxide support modification, while improving the mass electrochemical activity of the precious active components. Another feasible way is by enhancing the catalytic sites utilization, which is possible to achieve by interface engineering, involving both the interface between the membrane and the catalyst layer to promote the ionic conductance and the interface between the catalyst layer and porous diffusion layer to promote the electronic conductance.

2) For the porous CCS‐based electrodes commonly used in AEMWEs, the mild alkaline operating condition makes a variety of non‐precious electrocatalysts applicable, for example, unitary or binary transition metal oxides, LDHs, and perovskites, and so on. 3D metal‐based self‐supporting porous electrodes with relatively thick catalyst layers and rich pores are emerging as the most promising candidates. Various preparation strategies, including electrochemical deposition, hydrothermal growth, impregnation, and corrosion engineering have been explored for fabricating the 3D self‐supporting porous electrodes. Compared with the traditional CCS‐based electrodes prepared by the spray coating or dip coating method, this kind of self‐supporting electrode is featured with highly exposed active surface area and abundant hierarchical pores to ensure sufficient catalytic sites and mass transportation especially for high‐current reactions. Besides, the ionic network construction and the surface wettability/hydrophobicity optimization are also concerned to benefit the electrode reaction on 3D self‐supporting porous electrodes.

3) Direct seawater electrolysis can be achieved based on different membrane electrolyzers via electrode engineering. By creating a preferential OH^−^‐enriched microenvironment on the electrode surface, it is possible to inhibit chlorine oxidation by hindering the approach of chloride ions to the catalyst surface in the PEMWE feeding with filtered seawater. Hybrid electrolysis based on the alkaline environment in the AEMWE and the cation exchange membrane electrolyzer can be utilized for direct seawater electrolysis, with reduced overpotential by replacing OER with thermodynamically more favorable oxidation reactions (e.g., HzOR and SOR), and optimized electrode surface properties to prevent chlorine oxidation and corrosion. Besides, by applying a waterproof and breathable PTFE membrane, direct seawater electrolysis could be achieved by ensuring a self‐dampening electrolyte with the changed water migration way through the gas‐path interface of the PTFE membrane.

Despite significant progress has been achieved with electrode engineering in membrane electrolyzers from these aspects as reviewed in this work; we believe that membrane‐based electrode engineering will be a dynamic new research field with extensive scientific significance in the future. From one prospect, with the advancement of membrane technologies, for example, the advent of polymer exchange membranes that can withstand operating temperatures slightly higher than 100°C,^[^ ^144^, ^145^ ^]^ electrode engineering toward building hydrophobic/aerophilic electrode surfaces can be expected to achieve more‐efficient water vapor electrolysis, and apply precious metal free electrocatalysts that are sensitive to the acidic OER with the existence of liquid water. From another prospect, more attention could be paid to the improvement of the HER cathode in water electrolyzers. At present, research interests have been largely focused on optimizing the OER anode, aiming to improve the overall electric efficiency of water electrolyzers and reduce the cost. While for the HER cathode, Pt/C catalysts with high precious metal proportions are still widely used. By developing more stable and effective Pt‐free cathode materials and structures,^[^ ^146^, ^147^ ^]^ it can be expected to further reduce the hydrogen production cost in water electrolyzers. In the future, electrode engineering is also likely to focus more on water electrolysis dealing with extensive sources of water, like seawater, and other accessible freshwater resources, such as lake water, river water, tap water, and so on with higher economic benefits. This aspect is likely to be realized by endowing electrode selectivity, or by introducing an in situ water purification pathway.

## CONFLICT OF INTEREST STATEMENT

Zongping Shao is a member of the *Exploration* editorial board. The authors declare no conflict of interest.
